# Spatial distribution of African Animal Trypanosomiasis in Suba and Teso districts in Western Kenya

**DOI:** 10.1186/1756-0500-3-6

**Published:** 2010-01-15

**Authors:** Samuel M Thumbi, Joseph O Jung'a, Reuben O Mosi, Francis A McOdimba

**Affiliations:** 1 Centre for Infectious Diseases, School of Biological Sciences, University of Edinburgh, Kings Buildings, West Mains Road, Edinburgh, EH9 3JT, UK; 2 International Livestock Research Institute, P.O Box 30709-00100, Old Naivasha Road, Nairobi, Kenya; 3 Department of Animal Production, Faculty of Veterinary Medicine, University of Nairobi, Kenya. P.O Box 29053-00100 Nairobi; 4 Institute of Primate Research P.O Box 24481, 00502 Nairobi, Kenya; 5 Department of Pathology, Aga Khan University Hospital, P.O Box 30270-00100 Nairobi, Kenya

## Abstract

**Background:**

Studies on the epidemiology of African Animal Trypanosomiasis (AAT) rarely consider the spatial dimension of disease prevalence. This problem is confounded by use of parasitological diagnostic methods of low sensitivity in field surveys. Here we report a study combining highly sensitive and species specific molecular diagnostic methods, and Geographical information system (GIS) for spatial analysis of trypanosome infection patterns, to better understand its epidemiology. Blood samples from 44 and 59 animals randomly selected from Teso and Suba districts respectively were screened for trypanosomes using PCR diagnostic assays. Spatial distribution of the positive cases was mapped and average nearest neighbour analysis used to determine the spatial pattern of trypanosome cases detected.

**Findings:**

Trypanosome prevalence of 41% and 29% in Suba and Teso districts respectively was observed. *T. vivax *infections were most prevalent in both areas. Higher proportions of *T. brucei *infections (12%) were observed in Suba, a known sleeping sickness foci compared with 2% in Teso. Average nearest neighbour analysis showed the pattern of trypanosome infections as random. An overlay with tsetse maps showed cases lying outside the tsetse infested areas, mostly being cases of *T. vivax *which is known to be transmitted both biologically by tsetse and mechanically by biting flies.

**Conclusion:**

These findings suggest a need to design control strategies that target not just the biological vector tsetse, but also the parasite in cattle in order to clear the possibly mechanically transmitted *T. vivax *infections. There is need to also review the accuracy of available tsetse maps.

## Findings

Trypanosomiasis, a disease of humans and animals caused by several species of trypanosomes and spread by tsetse flies is a major constraint to livestock production in 37 countries within the Sub-Saharan region. An estimated 45-50 million cattle are at risk of infection in the region, with an estimated economic loss of up to US$ 1.3 billion in cattle production [1]. Its public health importance has led to attempts to control the disease nationally and regionally with initiatives as Pan Africa tsetse and trypanosomosis eradication program (PATTEC) [2]. These attempts rely on repeated large-scale epidemiological studies and environmental surveys, guiding the design and implementation of intervention strategies. The accuracy of these surveys is limited by use of parasitological diagnostic techniques as microscopy due to low sensitivity [3], and the difficulty in incorporating climatic and environmental data known to influence tsetse distribution, and as a result disease spread [4,5].

The high costs required to produce tsetse distribution maps through ground-based vector surveys have resulted in few studies looking at the spatial dimension of disease prevalence [6]. However, the use Geographical Information System (GIS) software now makes it cheaper and easier to produce maps which can serve as useful tools for policy discussion, and allow for analysis of factors that would influence the disease patterns [7]. Polymerase Chain Reaction (PCR) diagnostic assays overcome the low sensitivity limitations of parasitological techniques. They are powerful tools for identification and diagnosis of trypanosomes in their hosts and vectors although their high cost and need for elaborate expertise has delayed their adoption [8]. However, PCR assays able to detect all pathogenic trypanosome species in a single reaction have been developed [9,10]. These reduce the costs of screening a sample from an endemic area by up to five times, and have been suggested as suitable for large-scale epidemiological studies [11].

Results obtained from molecular studies, associated with geo-referenced information concerning vector, cattle distribution and relevant environment parameters, combined in a GIS have the potential of providing more informative study results. This new approach of studying complex pathogenic system is argued to lead to a better evaluation of the risk of infection, allows for effective risk communication, and gives scientific outputs in ways that are understandable to non-scientists [12]. Here we report a study combining use of highly sensitive and species specific PCR assays, with GIS for spatial analysis of infection patterns. The aim is to provide information about the prevalence and distribution AAT in two areas in Western Kenya, and suggest improved control strategies based on the findings.

## Results

### Cross-sectional trypanosomiasis survey

Overall, 29% of the 44 animals and 41% of the 59 animals sampled in Teso and Suba districts respectively had trypanosome infections. Based on the total number of cases detected, *T. vivax *infections were predominant in both areas (69% in Teso, and 50% in Suba). In Teso, infections with *T. congolense *savannah and *T. brucei *showed equal proportions of 8% while *T. congolense *savannah/*T. vivax *mixed infections made up 15% of the cases. In Suba, *T. brucei *made 17% and *T. congolense *savannah 8% of the cases. Mixed infections between *T. congolense *savannah and *T. vivax *made up for 12%, between *T. congolense *savannah and *T. brucei *infections 8%, and between *T. vivax *and *T. brucei *infections 4%. The prevalence of each of the trypanosome species in the two districts are shown in Table 1. Suba district showed a higher prevalence for *T. brucei *at 7% compared with Teso at 3%. The mixed infections observed in the two study areas are presented in Table 1. None of the *T. brucei *tested positive for the Serum Resistance Associated (SRA) gene, present only in the human-infective *T. brucei *rhodesiense.

**Table 1:** The prevalence of different species of trypanosomes in Teso and Suba districts of Western Kenya

	Prevalence in %			
Trypanosome species	Suba	95% CI	Teso	95% CI
TCS	3	(0 to 13)	2	(0 to 13)
T.vivax	20	(11 to 33)	20	(10 to 35)
T.brucei	7	(2 to 17)	2	(0 to 13)
TCS/T.vivax	5	(1 to 15)	5	(0 to 17)
TCS/T.brucei	3	(0 to 13)	0	-
T.vivax/T.brucei	2	(0 to 10)	0	-

Total	41	(28 to 54)	29	(17 to 45)

TCS - *T. congolense *savannah

### Spatial pattern of the disease

**Figure 1:**
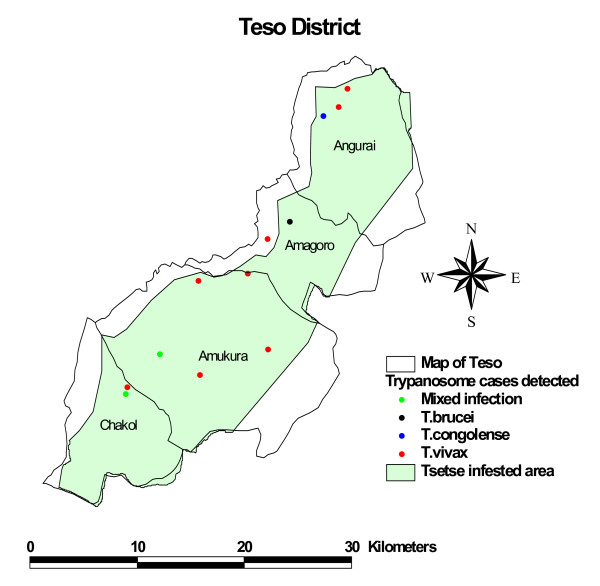
**Map of Teso district showing the areas infested with tsetse flies (grey), and the different species of trypanosomes detected**.

**Figure 2:**
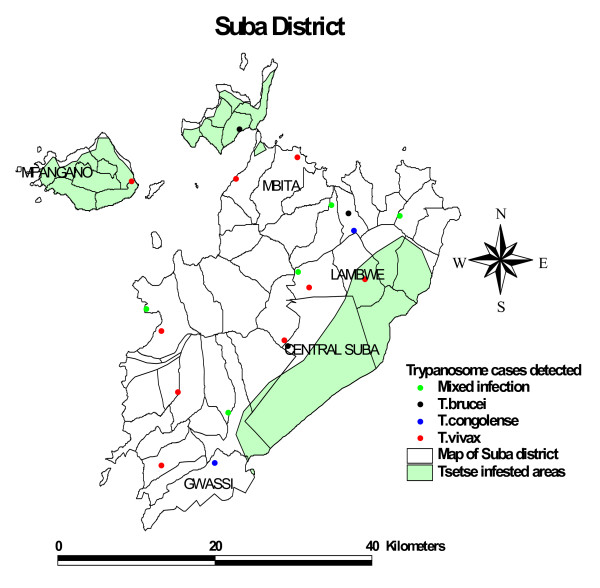
**Map of Suba district showing the areas infested with tsetse flies (grey), and the different species of trypanosomes detected**.

Figures 1 and 2 are maps showing the various trypanosome species detected and the overlay with the tsetse distribution maps for Teso and Suba districts respectively. The results show infection cases falling in the tsetse free areas. Most of these are cases of *T. vivax *which is known to also be transmitted mechanically through biting flies besides by tsetse. The ratio of the observed mean distance versus expected mean difference was 1.1 and 0.85, and the Z score 0.9 and -1 standard deviations, in Suba and Teso districts respectively. Z-score value is considered significant for dispersion at the confidence level of 0.01 if it is 2.58 or higher. Infection patterns in both Teso and Suba were random.

## Discussion

Attempts to control trypanosomiasis are based on large-scale epidemiological studies, active detection and treatment of confirmed cases, combined with tsetse control programmes [2]. The accuracy of these studies depends on the quality of the study design, and on the sensitivity and specificity of diagnostic methods used. Previous studies have for instance reported *T. congolense *as the predominant species in cattle in various parts of Africa [13,14], whereas this study established *T. vivax *as the most prevalent species. This finding agrees with other studies conducted in Busia district, Kenya [15], and in Tororo, South East Uganda [16]. *T. vivax *is pathogenic and sometimes causes a fatal hemorrhagic disease.

Overlays with the tsetse maps showed a large proportion of trypanosome cases falling in tsetse-free areas. Assuming the maps are accurate, we suspect that the transmission in those areas is mechanical since most infections are *T. vivax*, which is transmitted both by tsetse flies and mechanically by biting flies. A longitudinal study comparing bovine trypanosomiasis in tsetse free and tsetse infested areas of northwest Ethiopia found *T. vivax *to be responsible for 90.9% of the cattle trypanosome cases in the tsetse free zone support this hypothesis [17]. Animals may move between the tsetse-infested and tsetse-free areas although this is unlikely to adequately explain the high infection numbers in tsetse free areas. Farmers in the study region either tether their animals or graze them communally within limited areas.

The presence of mixed infections emphasizes the need for epidemiologists to focus beyond single species to multiple infections with different parasites which are a norm under field conditions [18]. In addition to trypanosomes, animals used in this study may have been infected with other parasites endemic in the region.

Lower trypanosome prevalence in Teso compared with Suba district could be attributed to the fact that unlike Suba, Teso was part of the FITCA program which focused on tsetse control and animal treatment in selected regions [19]. The small sample sizes used in this study limit its power to adequately detect true differences in the two study districts. A similar study using larger sample sizes is recommended. In Teso, a temporal decline of trypanosome prevalence is reported, with up to 40% prevalence in the late 90's, [20] and only 4% in 2005 [15]. Our study reports a much higher prevalence (29%) possibly reflecting higher sensitivity levels attained while using the PCR assays, or a possible lapse in stringent control measures following cessation of the FITCA campaigns. This apparent resurgence raises a question on sustainability of such control programs after donors pull out. Large differences in prevalence as may be obtained by PCR and parasitological methods could have a significant impact on the control strategies selected.

The tsetse maps used here are dated the year 1998, and land usage could have interfered with the tsetse habitat and hence it's distribution. For instance, in Teso district, the status of tsetse may have changed following the FITCA campaigns. Use of impregnated targets and deltamethrin treated cattle has been shown to reduce tsetse populations by 90% and the incidence of bovine trypanosomiasis from over 30% to below 5% in a 6 month period [21].

Whereas this study was not designed to identify the spatial risk factors associated with trypanosomiasis, it shows the importance of simple mapping in helping understand the status of the disease, and possibly guiding future research questions and policy formulation in disease mitigation strategies. For instance, is mechanical transmission of trypanosomiasis significant or is it a case of tsetse habitats having extended to areas captured as tsetse free before? A combination of highly sensitive diagnostic methods which can be extended to study infection in the vector tsetse and GIS provides an opportunity to use evidence based control strategies.

## Conclusion

The prevalence maps correlated with tsetse maps show cases lying outside the tsetse areas, mostly being cases of *T. vivax *which is known to be transmitted both biologically by tsetse flies and mechanically by biting flies. These findings suggest that control of tsetse is not enough and that to clear the possibly mechanically transmitted *T. vivax *infections, trypanosomes in cattle should also be targeted.

## Methods

### Study area

The cross-sectional study was carried out between August and September 2006 in Teso and Suba districts of Western Kenya. Both districts fall within the tsetse belt and are endemic for trypanosomiasis. Teso district lying towards north and bordering Uganda had recently been involved in a tsetse control program (Farming in tsetse controlled areas -FITCA), that run from the year 2000 to 2004. Suba district lying towards the south was not part of the FITCA program. Lambwe valley a known sleeping sickness foci, is within Suba district. This region offers a good environment for tsetse flies and wildlife which are the major reservoirs for Human African Trypanosomiasis. This study aims at establishing the prevalence of trypanosomiasis in the two districts, giving a possible indication of the impact of the tsetse control program.

### Blood sampling, DNA extraction

A total of 44 and 59 blood samples were obtained from Teso and Suba districts respectively. A multi-stage sampling plan was used. The first was sub-location selection in which all sub-locations in each district were allocated a unique number and a random sample picked. The farms in each sub-location were randomly selected based on a frame supplied by the respective local veterinary office. At the farm level one or two animals were randomly selected depending on availability and total number of animals in each farm. Indigenous cattle of both sexes and all ages except suckling calves were sampled. Genomic DNA for the screening of trypanosomes was extracted from the blood samples using the salt out method [22] with slight modification.

### PCR Diagnosis

Samples were screened for pathogenic *T. congolense*, *T. vivax *and *T. brucei *species. Species specific primers [23], and multi-species detecting ITS1 BR and ITS1 CF primers [9], and nested ITS 1, 2, 3 and 4 primers [10] designed to amplify the Internal transcribed spacer of the ribosomal DNA were used to screen each sample. The details of these screenings are well described in a previous paper by the same authors [11]. All *T. brucei *positives were further tested for the serum resistance associated (SRA) gene, which is known to be present only in the human infective *T. brucei *rhodesiense [24,25].

### Spatial distribution and analysis

Using the geographical coordinates of sampling areas, and results from the PCR screenings, infection distribution maps for each study site were created. Spatial pattern of infections were determined using the average nearest neighbour analysis feature in ESRI ArcGis 9.0. According to the software's manual, this is done by calculating the average distance between each feature and it's nearest neighbouring feature within the dataset. The result is the observed mean distance which is then divided by the expected mean distance to calculate the average nearest neighbour ratio. This value is compared with the average distance value for a hypothetical random distribution containing the same number of features and area. If the average nearest neighbour ratio or index is equal to 1, the pattern is random, a ratio greater than 1 indicates, the pattern is dispersed, while a ratio less than 1 implies the pattern is clustered.

### Tsetse distribution

Additional data layers describing the tsetse distribution in the two study areas were obtained from a GIS database available at the International Livestock Research Institute website http://www.ilri.org/GIS. The original map was based on tsetse distribution maps by Ford and Katondo [26], modified using data generated by Kenya Trypanosomiasis Research Institute. These tsetse distribution maps were overlaid on the trypanosome infection maps to determine whether all cases fell within tsetse infested areas.

## Competing interests

The authors declare that they have no competing interests.

## Authors' contributions

SMT drafted the manuscript. SMT, JOJ, FAM and ROM conceived the study. FAM and SMT carried out the molecular and spatial analysis. All authors read and approved the final manuscript.
